# Introduction of Otolaryngology Outpatient Examination Training Program for junior residents as part of rural regional medical support in Japan

**DOI:** 10.1002/jgf2.565

**Published:** 2022-08-04

**Authors:** Takeshi Watanabe, Hayato Takayama, Hisayuki Hamada, Kenichi Kaneko, Kayoko Matsushima, Atsuko Nagatani

**Affiliations:** ^1^ Department of Regional Medical Support Center Nagasaki University Hospital Nagasaki Japan; ^2^ Department of Medical Education Development Center Nagasaki University Hospital Nagasaki Japan; ^3^ Regional Medical Resources Support Center in Nagasaki Nagasaki Japan; ^4^ Nagasaki Prefecture Kamigoto Hospital Nagasaki Japan; ^5^ Department of Emergency Medical Education Center Nagasaki University Hospital Nagasaki Japan; ^6^ Watanabe ENT clinic Nagasaki Japan

**Keywords:** otolaryngology outpatient training program, residents, rural island hospital

## Abstract

**Background:**

Nagasaki Prefecture is located in the most western part of Japan, and there are a considerable number of clinics in its many remote islands and rural areas. Thus, the Regional Medical Support Center in Nagasaki Prefecture dispatches doctors to rural hospitals to provide medical support. We introduced an outpatient training program at these rural hospitals for all residents to improve their clinical training in the field of otorhinolaryngology, whereby one otolaryngologist trains one resident.

**Methods:**

This otolaryngology outpatient training program is randomly assigned, and conducted for 4–5 days a year, transported by a helicopter in Nagasaki Prefecture, which is a 30‐minute one‐way trip. We used a case checklist that included the 35 items that should be experienced and are defined as frequent by the Ministry of Health, Labor and Welfare. We also conducted a survey using an anonymous questionnaire.

**Results:**

The survey response rate was 100%. Comparing the experience rate of symptoms between the pre‐introduction resident and the post‐introduction resident who underwent the otolaryngology outpatient training program, the experience rates of common diseases, including vertigo and otolaryngologic symptoms such as nasal bleeding and hoarseness, significantly increased after the program was introduced (*p* ≤ .001). Notably, the experience rate of headache, cough/sputum, and vertigo was 100%.

**Conclusion:**

Our training program provides a suitable medical environment for the resident and secures a doctor who can provide secondary medical service support. Furthermore, the program will improve the level of primary care provided by the residents in remote island and rural area hospitals.

## Graphical abstract

We introduced an outpatient training program at these rural hospitals for all residents to improve their clinical training in the field of otorhinolaryngology, whereby one otolaryngologist trains one resident. Our training program provides a suitable medical environment for the resident and secures a doctor who can provide secondary medical service support. Furthermore, the program will improve the level of primary care provided by the residents in remote island and rural area hospitals.

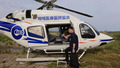

## 1 INTRODUCTION

**FIGURE 1:**
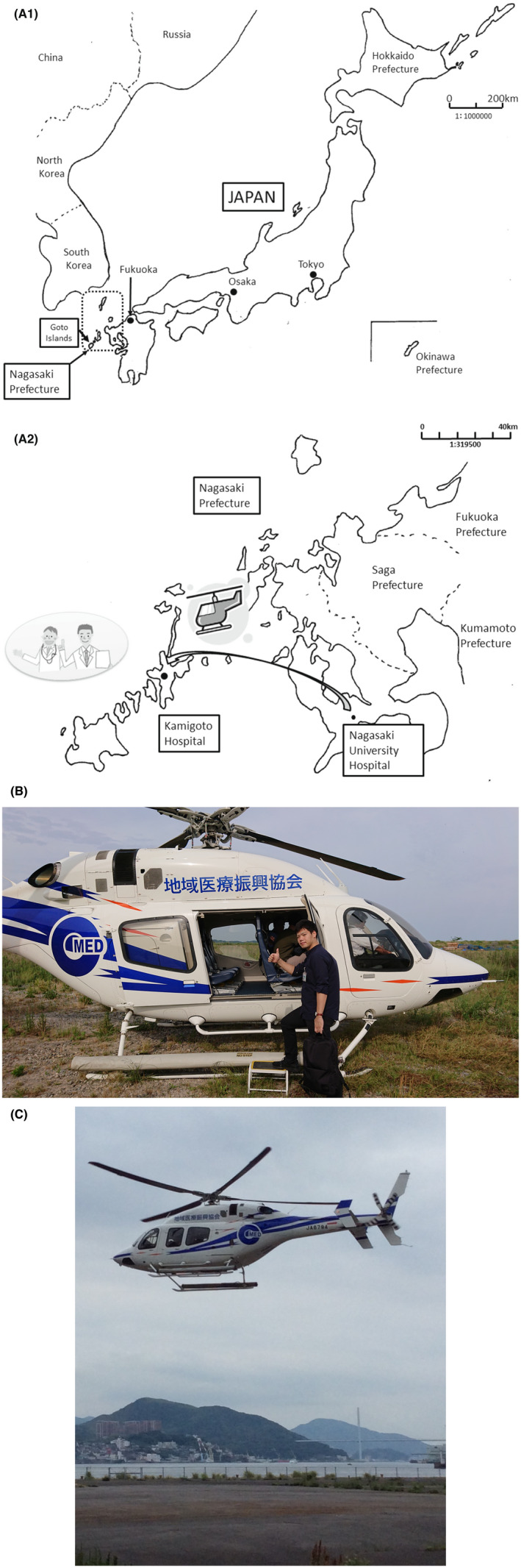
Nagasaki Prefecture is located in the westernmost part of Japan, and the Goto Islands are not connected by land, so doctors are dispatched by helicopter. (A1) Map of Japan showing the location of Nagasaki Prefecture. (Original) (A2) Locational relationship between Nagasaki University Hospital and Kamigoto Hospital. (Original) (B) A doctor on board a helicopter for transportation between Nagasaki University Hospital and Kamigoto Hospital. (C) Takeoff of a helicopter carrying doctors

Nagasaki Prefecture is located in the most western part of Japan (Figure 1A), and there is a total of 63 clinics in its many remote islands and rural areas. Doctors are dispatched by the Nagasaki Regional Medical Support Center to these rural area clinics as part of regional medical support to perform substitute medical services, such as the treatment of sudden illness, and business trips. Since 2012, Nagasaki University Hospital has introduced outpatient training at remote island and rural area hospitals for all residents to enhance clinical training and increase awareness of medical care in these areas. In this outpatient training program, one instructor doctor and one resident doctor visit a designated remote island or rural area hospital to perform outpatient training. Hynes et al. reported a model for ambulatory education and a feedback curriculum for residents.^1^ Furthermore, many reports have shown that outpatient training for junior residents was very effective, but these focused on internal medicine.^2^, ^3^, ^4^, ^5^, ^6^ Since 2014, we have incorporated an otolaryngologist instructor into our training program and are conducting outpatient training at Kamigoto Hospital, a remote island hospital in Nagasaki Prefecture. In addition to introducing an otolaryngology component to the outpatient training program, we administered a questionnaire survey to the junior residents to determine whether the introduction of outpatient training in otolaryngology affected the detection rate of symptoms, which is one of the goals of the clinical training for doctors set by the Ministry of Health, Labor and Welfare. The otolaryngology outpatient department of Kamigoto Hospital in the Nagasaki prefecture sees patients with many primary otolaryngologic diseases (vertigo or pharyngitis). Appropriate facilities and equipment are in place, and there is adequate space for medical examinations and procedures. Furthermore, the patients are very supportive of medical care, and there are no complaints about the performance of examinations by the resident doctors. There is one otolaryngologist at Kamigoto Hospital, and there is no bias in outpatient diseases.

## 2 METHODS

This study was a statement that the subject gave informed consent and patient anonymity were preserved. This otolaryngology outpatient training program was conducted in the otolaryngology outpatient examination room at Kamigoto Hospital in Shin‐kamigoto town, Nagasaki Prefecture. Kamigoto Island is approximately 100 km from Nagasaki city (Figure 1B:This medical staff has given consent to publish the photo.). Thus, we transport patients to Nagasaki, using a helicopter operated by the Nagasaki Prefecture Regional Promotion Association for Transportation (Figure 1B–D), which is a 30‐min one‐way trip. The outpatient training is randomly assigned, and it is conducted for 4–5 days a year.

There are 35 items that should be experienced and are defined as frequent by the Ministry of Health, Labor and Welfare (Table 1). We enrolled 113 junior residents in our outpatient training program at Nagasaki University Hospital before the introduction of otolaryngology outpatient training, and 152 junior residents enrolled after that. During the training, the residents should be able to experience at least 20 items, including nasal bleeding (epistaxis), hoarseness, hearing loss (of otolaryngologic origin), vertigo, cough/sputum, fever, headache, nausea/vomiting, dysphagia, and lymphadenopathy. We compared the experience rate before and after introducing the otolaryngology training program. We used a case checklist that included the 35 items that should be experienced and are defined as frequent by the Ministry of Health, Labor and Welfare. We also conducted a survey using an anonymous questionnaire. The questionnaire was performed immediately after this training program. We performed statistical analysis using Pearson's chi‐squared test.

**TABLE 1:** Frequent symptoms to be experienced as a goal of the doctor clinical training set by the Ministry of Health, Labor and Welfare

1. General fatigue
2. Insomnia
3. Anorexia
4. Weight loss
5. Edema
6. Lymphadenopathy
7. Rash
8. Jaundice
9. Fever up
10. Headache
11. Vertigo
12. Syncope
13. Cramps
14. Visual impairment/field narrowing
15. Conjunctival congestion
16. Hearing impairment
17. Epistaxis
18. Hoarseness
19. Chest pain
20. Palpitations
21. Dyspnea
22. Cough /Sputum
23. Nausea/Vomiting
24. Heartburn
25. Dysphagia
26. Stomach pain
27. Diarrhea/Constipation
28. Back pain
29. Joint pain
30. Walking disorder
31. Numbness in limbs
32. Hematuria
33. Dysuria
34. Abnormal urine output
35. Anxiety and depression

*Note:* Underlined symptoms are those that may be experienced by otolaryngology examination training.

**FIGURE 2:**
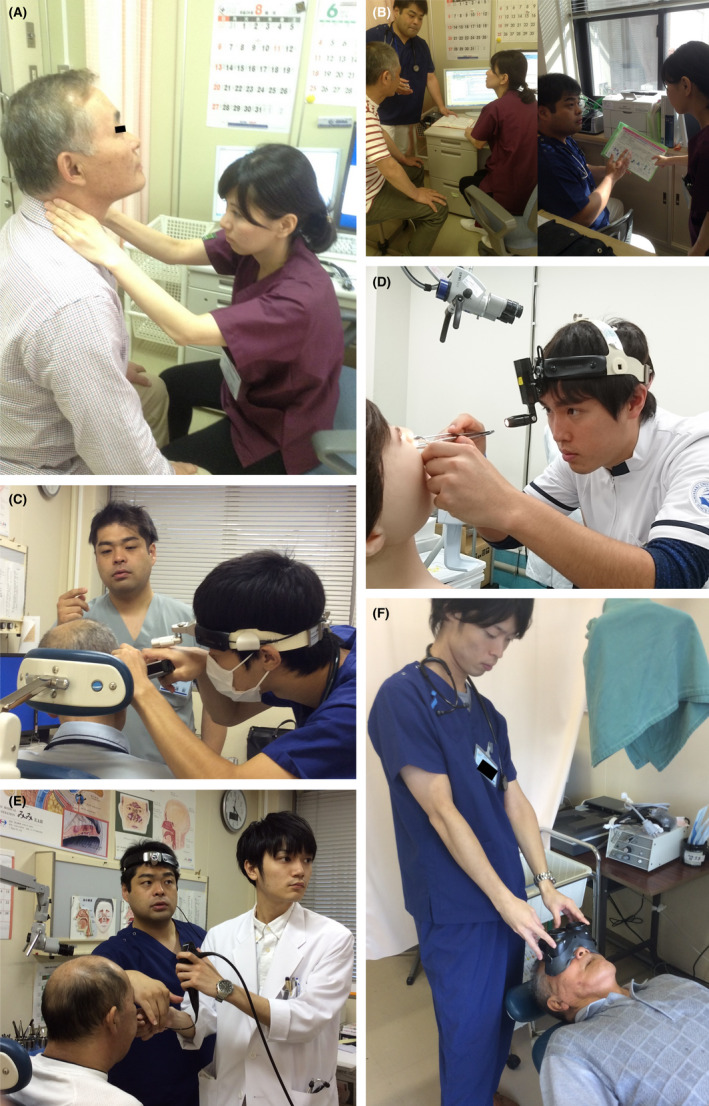
Outpatient training program for otolaryngology at Nagasaki University Hospital. Medical examination room with a patient undergoing an interview and consultation by the junior resident alone. (A) Consideration of the treatment policy with the instructor. (B) Otolaryngology examination training scene with the resident and the instructor giving the resident experience in (C) eardrum observation using a handheld otoscope, (D) epistaxis coagulation, (E) laryngoendoscopy, and (F) vertigo examination using Frenzel spectacles

To conduct the outpatient training, we selected a new outpatient and assigned them to the examination room. Then, the resident conducted a medical examination and consultation on their own and formulated their own medical policy. Thereafter, they presented the case and consultation results to the instructing doctor and reconsidered their medical policy. If necessary, the resident and the instructor re‐examined the patient and repeated the procedures. We retrospectively evaluated this flow of examination and the feedback from all residents in the training otolaryngology program (Figure 2: Their medical staffs have given consent to publish the photo).

## 3 RESULTS

The survey response rate was 100%. Comparing the experience rate of symptoms between the pre‐introduction resident and the post‐introduction resident who underwent the otolaryngology outpatient training program, the experience of original ear, nose, and throat (ENT) symptoms experience rate(%) increased, such as epitaxis from 0% to 19%, hearing impairment from 4% to 22%, and hoarseness from 8% to 30%. Additionally, there was an increase in the experience of the so‐called common diseases and original ear, nose, and throat (ENT) symptoms experience rate(%) of vertigo (55% to 100%), fever (79% to 100%), cough/sputum (91% to 100%), headache (71% to 95%), lymphadenopathy (19% to 43%), dysphagia (18% to 43%), rash (14% to 44%), conjunctival congestion (6% to 22%) and general fatigue (69% to 95%). Statistically significant difference for those above all the items are *p* ≤ .0001.

Thus, the training program improved the experience rate. Notably, the experience rate of headache, cough/sputum, and vertigo was 100% (Table 2). In addition, “rash”, “walking disorder” and “visual impairment” are related to otolaryngology. The “rash” is related to ear‐nose‐throat (ENT), such as ear eczema, especially Ramsay Hunt syndrome, nasovestibular eczema, lip eczema, and so on. The “walking disorder” is related to vertigo. The “visual impairment” is caused by rhinogenic optic neuropathy from acute sinusitis.

**TABLE 2:** Comparison of experience numbers and rates following the introduction of otolaryngology outpatient training

	Before	After	*p*‐Values
	*N*:152	*N*:113	
Epistaxis	0 (0%)	21 (19%)	*p* < .0001
Hearing impairment	6 (4%)	25 (22%)	*p* < .0001
Hoarseness	12 (8%)	34 (30%)	*p* < .0001
Vertigo	84 (55%)	113 (100%)	*p* < .0001
Fever	120 (79%)	113 (100%)	*p* < .0001
Cough/Sputum	138 (91°%)	113 (100%)	*p* < .0001
Headache	108 (71°%)	113 (100%)	*p* < .0001
Lymphadenopathy	29 (19%)	49 (43%)	*p* < .0001
Dysphagia	27 (18%)	49 (43%)	*p* < .0001
Rash	21 (14%)	50 (44%)	*p* < .0001
Conjunctival congestion	9 (6%)	25 (22%)	*p* < .0001
General fatigue	105 (69%)	107 (95%)	*p* < .0001

*Note: p* < .05: Significant difference.

When the other items were compared using a similar method, the experience rate was obviously improved for symptoms involving otolaryngology (Table. 2). There was no significant difference in the experience rate for symptoms that were not clinically related to otolaryngology.

Regarding the questionnaire results, the response to the open‐ended statement “I was glad to have the opportunity to see an ENT disease” included responses such as “When I was a medical student, I thought that the clinical range was narrow, but in reality, I thought that the clinical range was wide,” “It was useful to get the medical procedure and examination of epistaxis and vertigo,” and “Through the otolaryngology outpatient training, I gradually became interested in the otolaryngology department.”

## 4 DISCUSSION

Since the 1980s, the training of residents in Europe and the United States has become more important because of changes in medical care and insurance systems.^7^ On the other hand, in Japan, inpatient medical treatment is generally used as medical education for initial training, and outpatients are rarely used for medical education purposes. However, in the first‐visit outpatient examination in our training program, listening to the patient's complaint, taking note of physical findings, and selecting the necessary examination procedures within a limited time showed the positive effect of medical education for initial training. Because the residents were able to experience various diseases and symptoms, interest in this outpatient training system is increasing.^1^, ^2^, ^3^, ^4^, ^5^, ^6^ However, the current situation makes it challenging to implement effectively because of the scale of the designated hospital for training, the number of residents, and the number of instructors.

In 2020, the Ministry of Health, Labor and Welfare in Japan instituted outpatient training for junior residents. Nagasaki University Hospital introduced an outpatient training program for residents at remote island and rural area hospitals in 2012. Because of the vastly different settings of university hospitals compared with remote island or rural area hospitals, dispatching a doctor to these areas to train residents and support medical treatment results in more appropriate and effective training. In 2014, we introduced otolaryngology outpatient training in addition to our conventional internal medicine outpatient training to provide specialist medical care in otolaryngology to doctors of other departments as well as residents who are dispatched to remote island hospitals for training and eventually to patients themselves. This led to a noticeable improvement in the experience rate of epistaxis, hearing impairment, and hoarseness, which originate from otolaryngologic causes, as well as vertigo, fever, cough/sputum, headache, and lymphadenopathy. In particular, all residents experienced headache, cough/sputum, and vertigo. This indicates that the diseases and symptoms experienced in the ENT clinic are very closely related to the frequent symptoms that should be experienced during clinical doctor training as defined by the Ministry of Health, Labor and Welfare.

In other words, the goal of symptom experience by residents that is set by the Ministry of Health, Labor and Welfare, assumes that training in otolaryngology, especially outpatient training, is effective. The otolaryngology outpatient training at the remote island hospital of Kamigoto, which is carried out by this hospital, is significant because it allows the residents to become familiar with the otolaryngology department and learn about primary care. We also noticed an improvement in the image of the otolaryngology department from the responses to the open‐ended statement part of the questionnaire.

The ENT department is currently considered a minor medical department in Japan, and the number of doctors who have commenced specialized training as otolaryngologists in recent years is less than 250 annually, which is less than one‐tenth of those that train in internal medicine.^8^ Our training program dispatching a doctor to rural hospitals provides a suitable environment for the resident and secures a doctor who can provide secondary medical service support. Furthermore, the incorporation of such outpatient otolaryngology training will lead to improving the clinical skill of the junior residents.

## 5 CONCLUSION

The effectiveness of an outpatient training program by Nagasaki University Hospital to train junior residents in otolaryngology was revealed. The training program of dispatching a doctor to rural hospitals provides a suitable medical environment for the resident and secures a doctor who can provide secondary medical service support. Furthermore, the program will improve the clinical ability of residents to perform primary care.

## CONFLICT OF INTEREST

The authors have stated explicitly that there are no conflicts of interest in connection with this article.
